# Breast radiotherapy with kilovoltage photons and gold nanoparticles as radiosensitizer: An in vitro study

**DOI:** 10.1002/mp.15348

**Published:** 2021-12-01

**Authors:** Alessia Tudda, Elisabetta Donzelli, Gabriella Nicolini, Sara Semperboni, Mario Bossi, Guido Cavaletti, Roberta Castriconi, Paola Mangili, Antonella del Vecchio, Antonio Sarno, Giovanni Mettivier, Paolo Russo

**Affiliations:** ^1^ Department of Physics “Ettore Pancini” University of Naples Federico II Naples Italy; ^2^ INFN Division of Naples Naples Italy; ^3^ Medical Physics Department IRCCS San Raffaele Scientific Institute Milan Italy; ^4^ Department of Physics Specialty School of Medical Physics University of Milan Milan Italy; ^5^ Experimental Neurology Unit School of Medicine and Surgery University of Milano‐Bicocca Monza Italy; ^6^ INFN Division of Milano‐Bicocca Milan Italy; ^7^ INFN Division of Milan Milan Italy

**Keywords:** breast cancer, breast radiotherapy, gold nanoparticles, kilovoltage radiotherapy

## Abstract

**Purpose:**

We investigated the dose enhancement and internalization of gold nanoparticles (AuNPs) used as a radiosensitizer agent for rotational radiotherapy of breast cancer using a kilovoltage (kV) X‐ray beam.

**Methods:**

Human breast cancer cells MDA‐MB‐231 were incubated with or without 100 μg/mL (4.87 nM) or 200 μg/mL (9.74 nM) 15 nm AuNPs and irradiated with 100 kV, 190 kV, or 6 MV X‐rays. To assess the toxicity of the AuNPs, we performed a Sulforhodamine B assay. Using atomic absorption spectroscopy, scanning electron microscopy, transmission electron microscopy, and time‐lapse optical microscopy (rate of 2 frames per minute), we carried out a quantitative assessment of the amount of gold internalized by MDA‐MB‐231 cells and a characterization of the static and dynamical aspects of this internalization process.

**Results:**

No effect of AuNPs alone was shown on cell viability. Time‐lapse optical microscopy showed for the first time AuNPs cellular uptake and the dynamics of AuNPs internalization. Electron microscopy demonstrated AuNPs localization in endosomal vesicles, preferentially in the perinuclear region. After irradiation at doses up to 2 Gy, cell survival fraction curves showed increased mortality with AuNPs, with respect to irradiation without AuNPs. The highest effect of radioenhancement by AuNPs (at 9.74 nM AuNPs concentration) was observed at 190 kV showing a dose enhancement factor of 1.33 ± 0.06 (1.34 ± 0.02 at 100 kV), while at 6 MV it was 1.14 ± 0.06.

**Conclusions:**

The observed radio‐sensitization effect is promising for future radio‐enhanced kV radiotherapy of breast cancer and quantitatively in the order of previous observations for 15 nm AuNPs. These results of a significant dose enhancement were obtained at 15 nm AuNPs concentration as low as several nanomolar units, at dose levels typical of a single dose fraction in a radiotherapy session. Dynamical behavior of the 3D spatial distribution of 15 nm AuNPs outside the nucleus of single breast cancer cell was observed, with possible implications for future models of AuNPs sensitization.

## 1 INTRODUCTION

Breast cancer is the most common cancer in women, and the second cause of women's mortality.^1^ After early diagnosis by mammography screening, standard care requires tumor mass surgery, chemotherapy, and radiation therapy (RT) as adjuvant therapy. Conventional breast RT adopts megavoltage (6 MV) X‐ray beams, generated by a medical linear accelerator (linac). Radiation dose is delivered by two opposed MV tangential fields to encompass the breast, with possible irradiation of the chest wall and the lower axilla, with the patient in the supine position on the treatment coach. Conventional breast RT implies the delivery to the target volume of, for example, 45 to 50 Gy in 25 fractions of 1.8 to 2 Gy each, followed by a boost of 10 to 16 Gy, and is normally delivered in 6 to 6.5 weeks. This treatment unavoidably delivers substantial radiation dose to the lung, the heart (for left breast cancer), and carotid vessels, which might imply second‐cancer recurrences and metastasis and unacceptable normal tissue toxicity.^2^, ^3^ Hence, methods for reducing the dose to organs at risk are increasingly employed in breast RT, including intensity‐modulated radiotherapy, volume modulated arc therapy, and partial breast irradiation (PBI), as opposed to irradiating the whole breast, implemented by delivering high doses to the prone patient in a hypofractionated scheme. Stereotactic body radiation therapy (SBRT)—for example, using the 6 MV Cyberknife radiosurgery system—has been investigated, as well for breast RT, whereby high radiation doses are delivered in few fractions, and a cobalt‐60 unit for breast SBRT has been introduced recently.^4^

In this context, a line of research implied the use of metal particles in the sub‐100 nm size range, in particular gold nanoparticles (AuNPs), as effective radiation sensitizers in RT. The goal was to increase the dose to the tumor volume in RT, while keeping low the dose to normal tissues. This research was pursued actively by several groups in the last two decades, as reviewed in Schuemann et al. in 2016.^5^

Once injected in vivo, AuNPs are well tolerated and may concentrate preferentially in the tumor mass via the enhanced permeability and retention effect^6^, ^7^, ^8^, ^9^ in the highly vascularized tumor mass. The AuNPs, thus, accumulate in the tumor and enhance the local absorption of radiation, due to their high atomic number (Z_Au_ = 79), in a radio‐sensitization technique also known as dose‐enhanced RT.^10^ Production of reactive oxygen species (ROS) in normoxic cells and unbalance of the ROS homeostasis in hypoxic cells are possible causes explored to explain the observed radio‐sensitization effects.^11^, ^12^ Moreover, as a by‐effect of AuNPs injection, accumulation of AuNPs in tumor tissues may enhance the image contrast for image−guided RT. A pioneer study in vivo with mouse mammary subcutaneous tumors was carried out using AuNPs available commercially as X‐ray contrast agent for micro‐Computed Tomography (microCT).^7^, ^13^ The use of AuNPs as radiosensitizers from preclinical to clinical research has been reviewed recently,^14^, ^15^ illustrating possible mechanisms of physical, chemical, and biological phases of tumor tissue damage with AuNP‐induced enhanced toxicity.

AuNPs with sizes up to several tens of nanometers can be easily absorbed by cells and accumulate in tumor tissue, so acting as dose‐enhancement agents via their high photoabsorption. This enhancement phenomenon, observed for kV^6^, ^16^, ^17^, ^18^, ^19^ as well as for MV beams^6^, ^20^ and for proton beams,^21^ is understood as relying on the diffusion of the AuNPs in the tumor vasculature, and on the higher absorption of radiation in high‐*Z* particles localized close to radiation‐sensitive cellular sites. This effect is due to the short range of electrons produced by the absorption of (i) primary kV photons incident on the patient body or (ii) secondary kV photons and secondary electrons produced by MV primary X‐rays propagating in the tissue depth. Dose enhancement factors (*DEF*s) from 1.1 up to several units have been reported.^6^, ^16^, ^17^, ^18^ According to a recently published review exploring the steps needed for a clinical implementation of AuNPs‐enhanced RT,^5^ preclinical experimental results are (still) needed for studying in vitro biocompatibility and radiation sensitization effect, for future AuNPs‐based RT techniques.

On the other hand, a recently proposed technique for breast RT involves the use of collimated beam(s) from a kV X‐ray source rotating around the breast, with the patient in prone position and an under bed rotating gantry hosting an orthovoltage X‐ray tube with high filtration.^22^ This technique of kV external beam radiotherapy (kV‐ebRT) has raised some interest in breast RT and for lung cancer RT as well,^23^ and it appears well suited for combination with AuNPs dose enhancement procedures.

Recently, we started the project SR^3^T (Synchrotron Radiation Rotational RadioTherapy)^24^, ^25^, ^26^ for investigating breast RT using a kV photon beam and AuNPs dose enhancement. The proposed setup adopts a configuration in which the patients are in prone position with the breast hanging from a hole in the bed. Suitable collimation of the kV X‐ray beam in the horizontal and vertical planes—as well as rotation over 360° around a vertical axis passing through the tumor site coupled to horizontal translation of the axis of rotation in a rotate‐translate geometry—produces a PBI RT technique covering the entire tumor volume. Rotation of the beam around the breast at kV energies determines an accumulation of the absorbed dose along the axis of rotation, where the tumor is located. According to this phenomenon, a large skin‐sparing effect is produced with kV beams, analogous and quantitatively in the order of the one typical of MV radiotherapy due to the build‐up effect. One of the goals of the SR^3^T project is to investigate the use, during the treatment, of AuNPs injected in the patient at non‐toxic concentrations, as a radio‐sensitizing agent. Irradiation with kV X‐rays, with respect to MV X‐rays, permits to exploit the high value of the photoelectric cross‐section for AuNPs (concentrated in the cancerous cells). The presence of the gold nanoenhancers allows increasing in‐situ the absorbed dose.

This work reports on a series of experiments for observing the dose‐enhancement effect in a human breast cancer cell line incubated with commercially available AuNPs (15 and 1.9 nm diameter) at varying concentrations, after irradiation with kV and MV polyenergetic X‐ray beams. Experimental studies of AuNPs internalization have been carried out, also showing for the first time the dynamics of AuNPs internalization inside single cell at very low concentrations. The AuNPs dynamics observed might affect the radio‐sensitization effect, a future investigative goal prompted by the present experimental report.

## 2 MATERIALS AND METHODS

To evaluate the dose enhancement due to the effect induced by the accumulation of gold nanoparticles in cancer cells, we used a commercial AuNPs product, the AuroVist™ (Nanoprobe, Yaphank, NY, USA)^27^ consisting of AuNPs with a core diameter of 1.9 or 15 nm, stabilized with a highly water‐soluble organic shell. They were already used in small animal models for RT^7^, ^13^ and as contrast agent in X‐ray imaging studies.^28^, ^29^ In these models, they have shown low toxicity (lethal dose > 0.5 g Au/kg), low osmolality, and low viscosity (similar to water)^28^ and, therefore, they may be injected and used in small blood vessels without risk of vascular damage. To reach working concentrations, AuroVist™ was further diluted in cell culture medium.

### 2.1 Cell line and materials

All the experiments were performed on human breast cancer cell line MDA‐MB‐231, obtained from the American Type Culture Collection (ATCC, Manassas, VA, USA).^30^, ^31^ Cells were cultured in Dulbecco's Modified Eagle Medium (DMEM) supplemented with glutamine, antibiotics, and 10% fetal bovine serum (Euroclone, Pero, Italy). To evaluate the dose enhancement due to the effect induced by the accumulation of AuNPs in cancer cells, we used a commercial AuNPs product, the AuroVist™, with a core diameter of 1.9 or of 15 nm, purchased from Nanoprobe.^27^ They were dissolved at 200 mg/mL in sterile Phosphate Buffered Saline (PBS) and stored according to manufacturer's instructions. To reach working concentrations, AuroVist™ was further diluted in cell culture medium. All the other materials unless specified were from Merck Life Science S.r.l. (Milan, Italy).

### 2.2 Cell viability assay

MDA‐MB‐231 cells plated 1.5 × 10^4^/well in 96‐multiwell plates were treated with 50, 100, 200, and 400 μg/mL (2.43, 4.87, 9.74, and 19.48 nM, respectively) AuroVist™ 15 nm or with culture medium without AuroVist™ (control cells). Twenty‐four hours after AuroVist™ induction, Sulforhodamine B (SRB) assay was performed as elsewhere described.^32^ After SRB dye solubilization, optical density at 540 nm was measured using a multiplate reader (OmegaStar, BMG Labtech). Wells without cells but containing medium were assessed to account for unspecific staining and their value was subtracted as background. Proliferation was calculated as a percentage with respect to control cells using blank corrected values. Data are the results of three independent experiments (six replicates for each condition/experiment).

### 2.3 Atomic absorption spectroscopy

Quantification of AuNP in cells was performed as previously described in Ceresa et al.^33^ Briefly, cells were plated 2.5 × 10^5^ cells/well in six‐multiwell plates and treated for 24 h with 15 or 1.9 nm AuNPs (200 μg/mL) or with medium alone (control cells). Cell lysates were prepared and after three freezing/thawing cycles at −80°C, samples were vigorously vortexed. Samples were treated with highly pure HNO_3_ (Au ≤ 0.02 μg/kg, TraceSELECT Ultra, Sigma Chemical Co.) and Au content was measured by Graphite Furnace Atomic Absorption Spectroscopy (GF‐AAS) by using a Varian AA Duo graphite furnace atomic absorption spectrometer (Varian, Palo Alto, CA, USA) at the wavelength of 242.795 nm, as previously reported.^34^ The calibration curve was obtained using known concentrations of standard solutions purchased from Sigma Chemical Co. Au content was normalized by protein concentration in each sample.

### 2.4 Dynamics of AuNPs internalization

To study the dynamics of AuNPs internalization, live‐cell imaging was performed with NanoLive microscope (MEDIA System Lab, Italy), a holotomographic microscope that combines holography and rotational scanning enabling to measure the refractive index (RI) of the different parts of living cells without any staining. After image acquisition, the software STEVE is used to apply a quantitative staining based on the RI. Briefly, MDA‐MB‐231 cells were plated 2 × 10^5^ cells in a 35 mm glass‐bottom dish. The day after seeding, cells were treated with 15 nm AuroVist™ 200 μg/mL (9.7 nM) and live‐cell imaging began immediately after AuNPs induction. Holotomographic images were acquired every 30 s for 16 hours.

### 2.5 Scanning electron microscopy

MDA‐MB‐231 cells were plated 1.4 × 10^6^ in 60 mm dishes and treated for 24 h with 200 μg/mL (9.74 nM) AuroVist™ 15 nm. At the end of treatment, cells were fixed for 1 h in 4% paraformaldehyde and 2% glutaraldehyde, dehydrated in ethanol and embedded in epoxy resin. Scanning Electron Microscope (SEM) imaging was performed with a Volumescope System (Thermo Fischer Scientific, Waltham, MA, USA). Following *in‐situ* sectioning of the sample using a diamond knife (40 nm sections), the freshly exposed tissue was imaged several times using increasing accelerating voltages; images were subsequently processed with a deconvolution algorithm, obtaining isotropic datasets with less than 10 nm *z*‐resolution.

### 2.6 Transmission electron microscopy

MDA‐MB‐231 cells were plated 1.4 × 10^6^ in 60 mm dishes and treated for 24 h with 200 μg/mL (9.74 nM) AuroVist™ 15 nm or with complete medium without AuNPs (control cells). At the end of treatment, cells were fixed for 1 h in 4% paraformaldehyde and 2% glutaraldehyde. After 30 min postfixation in 1% OsO_4_ in cacodylate buffer, cells were dehydrated in ethanol and embedded in epoxy resin. Ultra‐thin sections (60 nm) were stained with uranyl acetate and lead citrate and observed with Philips CM10 transmission electron microscope (Philips Medical Systems S.p.A., Monza, Italy).

### 2.7 Clonogenic assay

To quantify the increase in dose absorption due to the presence of AuNPs, we performed a clonogenic assay. We evaluated the survival fraction at 2 Gy (*SF*_2_), the mean inactivation dose (*MID*) as the area under the survival fraction curve and Sensitizer Enhancement Ratio (*SER*) calculated by dividing the *MID* of nonexposed cells with respect to 15 nm AuNPs exposed cells, and the *DEF* ^34^ defined as the ratio between the radiation dose ratio of dose without AuNPs and the dose with AuNPs at a specific percentage of survival.^6^, ^35^

To perform the clonogenic assay, MDA‐MB‐231 plated 2.5 × 10^5^/well in six‐multiwell plates were treated with 100 μg/mL (4.87 nM) or 200 μg/mL (9.74 nM) AuroVist™ 15 nm or with culture medium without AuroVist™ (control cells). Twenty‐four hours after induction, the medium was removed and replaced with a fresh medium without AuroVist™. Cells were irradiated with 100 and 190 kV X‐rays, at doses of 1 and of 2 Gy using a Radgil X‐ray irradiator unit (Gilardoni, Mandello del Lario, LC, Italy), and 6 MV X‐rays using a Varian Clinac iX System linac. During irradiation, cells were maintained in PBS containing Ca^++^ and Mg^++^.

After irradiation, cells were harvested, counted, and plated in 100 mm dishes at low density (500 and 1000 cells/dish for not irradiated cultures, 1000 and 2000 cells/dishes for irradiated cultures) to obtain no overlapping detectable colonies (composed of at least 50 cells). Duplicates were plated for each density. After 14 days, culture dishes were stained with Crystal Violet. Pictures were taken and colonies counted by ImageJ freeware. Data are the results of three independent experiments (two replicates for each condition/experiment). For each experiment, the average value and the standard deviation of *SF* were calculated, and all the values out two‐sigma interval were excluded.

Radiation dose–response data were obtained by evaluating the surviving fraction (*SF*) of MDA‐MB‐231 cell cultures exposed to target doses (*D* = 1 Gy, 2 Gy) of X‐rays, using beams of three qualities (100 and 190 kV from an X‐ray irradiator, or 6 MV from a clinical linac). These doses are typical of single fraction dose values in conventional breast radiotherapy. *SF* data were expressed as mean ± standard error of triplicate measurements. These data were interpreted as following the linear–quadratic relationship expressed by the formula ln(*SF*) = ‐*aˑD* – *bˑD* ^2^. A unique quadratic interpolating function passes through the three data points (at 0, 1, and 2 Gy). As published,^35^ we also evaluated the *MID* as the area under the linear‐quadratic curves. The parameters *SF*_2_ (*SF* at 2 Gy), *MID*, and *DEF* have been used to intercompare radiation‐survival curves. At each beam quality, we calculated the *SER*, for 15 nm AuroVist A™uNPs, by dividing *MID* with no AuNPs present, by *MID* with AuNPs present, for each AuNP concentration (100 and 200 μg/mL).

### 2.8 Dose evaluation

We carried out absorbed dose measurements using GAFCHROMIC™ films (Ashland Specialty Ingredients G.P., Bridgewater, NJ, USA) type EBT3.^36^ EBT3 film are suitable for dose map measurements in radiation fields with high dose gradients, as in our measurements, because of the high spatial resolution and energy independence. To convert the response of the film into dose, we measured a calibration curve. For this purpose, we exposed *n* = 8 pieces of cut films (10 cm × 13 cm) to known different doses (0, 0.2, 0.4, 0.6, 0.8, 1, 1.2, 1.9 Gy), obtained by changing the exposure time. To ensure the stabilization of the polymers, 24 hours after irradiation, the films were scanned with an Epson Expression 11000XL scanner of San Raffaele Hospital (Milan, Italy) in transmission mode at a resolution of 72 dpi (0.35 mm pixel size). To obtain the calibration curve, we utilized the FilmQA™ Pro Software (Ashland Inc.), a quantitative analysis tool designed for film scanning, pixel value extraction, and dose conversion. This software works with a method called “triple channel evaluation,” which uses pixel values from all color channels together to construct the dose map, providing “corrected” red, green, and blue dose maps. It is also possible to separate the dose‐dependent part of the signal from the dose‐independent part, where the latter derives from nonhomogeneity related to film manufacturing, scanner artifacts, or film manipulation (dust, fingerprints).^37^ Practically, the method varies the dose values until the corresponding pixel values are best matched for all three‐color channels.

In the case of 6 MV irradiation, to plan the fields and beam arrangements for cell lines irradiations, the Medical Physics team of San Raffaele Hospital acquired a CT image of the experimental setup, then they imported it into the treatment planning system (TPS) *Eclipse* (Varian Medical Systems, Inc., USA) and simulated the irradiation session. To deliver a dose of 1 Gy to the cells, with an uncertainty of 5%, the TPS indicated the necessity to deliver 92 Monitor Unit (MU); for 2 Gy the TPS indicated a value of 184 MU, where MU measures the output of the clinical accelerator, measured by ionization chambers.^38^ The correct delivery of 1 and 2 Gy, respectively, was also checked placing a piece of EBT3 above the cell arrangement.

### 2.9 kV and MV X‐ray beams

**FIGURE 1:**
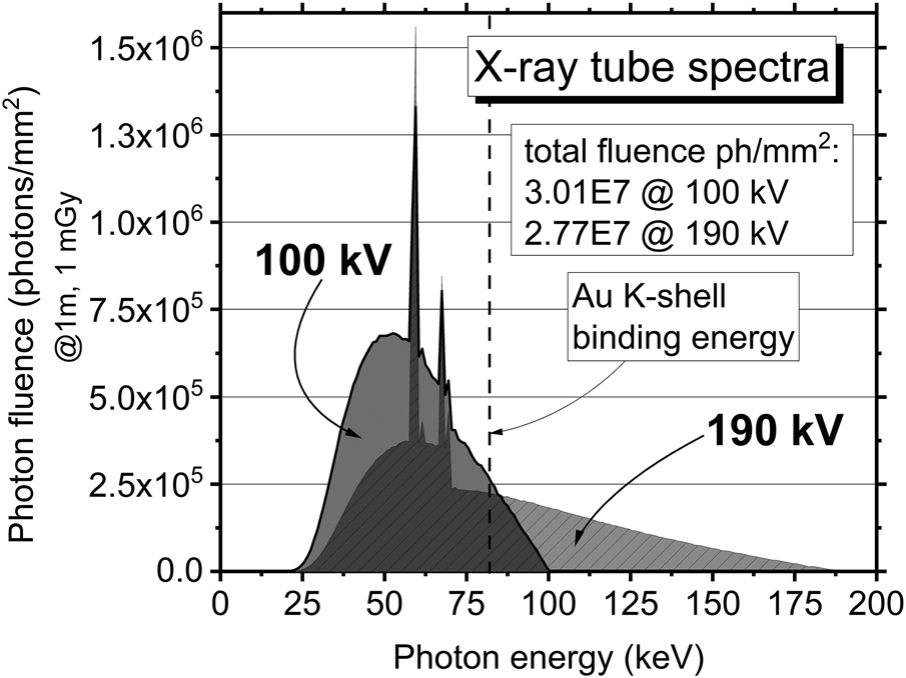
Photon fluence versus energy for the X‐ray beam (added filtration 0.3 mm Cu) at 100 and 190 kV (calculated HVL = 6.9 and 10.8 mmAl, respectively), at a fixed air kerma of 1 mGy at 1 m from the beam central axis. Data calculated with the code TASMICS^39^

The MDA‐MB‐231 cells were irradiated with kV beams with energies close to L‐ or K‐shell binding energies of Au, to have a high probability of generating photoelectrons via photoelectric absorption in AuNPs. The range of electrons in water does not exceed 140 μm for kinetic energies less than 100 keV (Continuous Slowing Down Approximation range < 2.5 μm for electron energy less than 10 keV). Hence, L‐shell photoelectrons have a range in water too short to exceed the size of a single cell. On the contrary, photons with energies higher than the K‐edge binding energy of Au (80.7 keV) produce photoelectrons that could deposit energy over a distance of four to eight cells from the interaction site. Hence, such a photon could produce a more uniform distribution of the absorbed dose over the tumor volume. On the other hand, the photon energy cannot be too high, because of the rapid decrease of the photoelectric cross‐section with photon energy. Based on these considerations, we decided to measure the *SF*_2_, *SER*, and *DEF* parameters for two different tube voltages: 100 and 190 kV. The spectra of these beams calculated using the TASMICS spectral model^39^ are shown in Figure 1. As regards the spectral quantity at low energies, a polyenergetic spectrum at 100 kV (average energy of 58 keV) could provide a greater probability for the emission of a photoelectrons of the Au L‐edge (L1 energy of 14.4 keV, L2 of 13.7 keV, L3 of 11.9 keV) than for a 190 kV beam: in that case, the photoelectron would have a maximum kinetic energy of approximately 43 keV. In the case of an Au L‐edge photoelectric event, the atomic de‐excitation via Auger emission is favored with respect to fluorescence emission (roughly by a fraction 3:1). In the case of the polyenergetic spectrum at 190 kV (average energy, 81 keV), a significant fraction of the photon spectrum is at energies higher than, and close to, the Au K‐edge (Figure 1) and may produce the ejection of a K‐shell photoelectron with kinetic energy of a few tens of keV. For example, for 135 keV photons interacting with K‐shell electrons in Au, a photoelectron of kinetic energy of about 55 keV would be produced, whose range in water is about 50 μm. To compare the results from kV beams with the X‐ray beam used in the conventional breast RT treatments, irradiations of a cell at 6‐MV clinical beam were performed at San Raffaele Hospital using a Varian Clinac iX™ System linac.

## 3 RESULTS

### 3.1 Quantification of Au internalization

Atomic Absorption Spectroscopy (AAS) was performed to quantitatively measure the amount of Au internalized by MDA‐MB231 cells. We compared the amount of Au present in cell lysates obtained from control untreated cultures and from MDA‐MB‐231 treated with 200 μg/mL of both 15 and 1.9 nm AuNPs. According to AAS, after 24 h of 15 nm AuNPs treatment in MDA‐MB‐231 cells the amount of Au was 1.66 μg/mg of protein content (range 0.53–3.35). Treatment with 1.9 nm AuNPs resulted in an Au amount of 0.16 μg/mg of protein (range 0.05–0.22). In control untreated cells, Au was not detectable in cell lysates. From these results, we decided to use only the 15 nm AuNPs in the following measurements.

### 3.2 Cell viability

**FIGURE 2:**
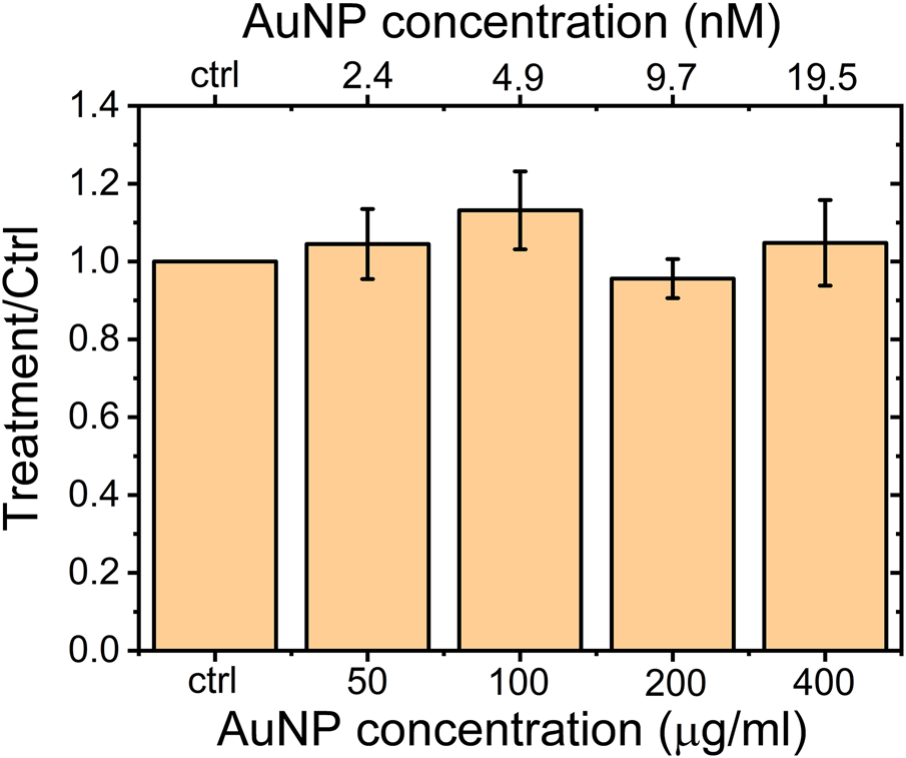
Evaluation of AuNPs toxicity on MDA‐MB‐231 cells. Bars represent mean values ± SD. No toxic effects are present up to 400 μg/mL

To investigate the toxicity of AuNPs on MDA‐MB‐231 cells, we assessed the effect of Aurovist 15 nm AuNPs on cell proliferation via the SRB assay. As shown in Figure 2 24 h‐treatment with any of the assayed 15 nm AuNPs concentrations (50–400 μg/mL) induced a statistically not significant modification of cell survival when compared to control untreated cells: this demonstrated that Aurovist™ is not toxic for MDA‐MB‐231 up to 400 μg/mL. As shown by Kanavi et al.,^8^ different kinds of cells have various radiation responses against RT at different concentrations of AuNPs. According to this observation and to our data two different AuNPs concentrations were used for subsequent experiments: Aurovist™ at 100 μg/mL (4.9 nM) and at 200 μg/mL (9.7 nM) were used for subsequent experiments.

### 3.3 AuNPs intracellular localization in MDA‐MB231 cells

**FIGURE 3:**
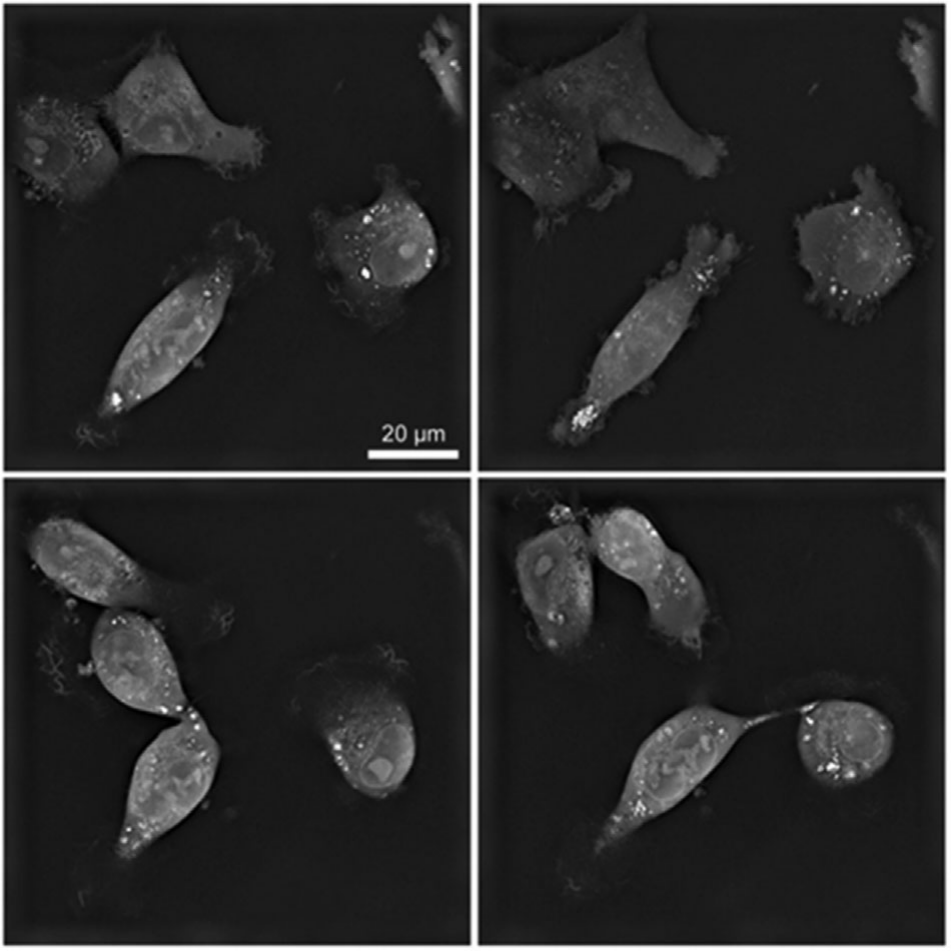
AuNPs internalization in MDA‐MB231 treated with 200 μg/mL AuroVist™ 15 nm. Each image refers to a single optical plane (thickness 200 nm) acquired during the acquisition by NanoLive holotomography. In these optical micrographies, AuNPs appear inside the cell as white clusters. These clusters are in the cytoplasm in structures like vesicles, and not in the nucleus.

Different morphological techniques were applied to study the intracellular localization of AuNPs in MDA‐MB231. First, we studied the dynamic process of Aurovist™ intake in living MDA‐MB‐231 cells treated with 200 μg/mL (9.7 nM) AuNPs 15 nm using Nanolive, a newly developed microscope that enables the visualization of subcellular structures based solely on their RI. Given the differences in refractive properties between cells and gold nanoparticles, the AuNPs are visualized as white structures, but it is not possible to detect AuNPs in the culture medium. Images were taken starting from Aurovist™ induction and up to 16 h observation time. The corresponding video, available in Supplementary Material (Video S1.avi), demonstrates that AuNPs were able to enter into the cells and move in the cytoplasm following cellular internal fluid flows. Differently sized nanoparticles clusters were present, distributing heterogeneously throughout the cells in the cytoplasm and in the perinuclear region, but not inside the nucleus, as confirmed by the representative frames pictured in Figure 3. Larger clusters of AuNPs were apparently enclosed in membrane delimited structures, probably vesicles or autophagosomes, which need further characterization. One of the cells represented in the video (on the left of single frame) underwent mitosis and the AuNPs were observed to split between daughter cells. One cell (on the right of single frame) underwent a wide cellular vacuolization, a clear sign of cellular stress.

**FIGURE 4:**
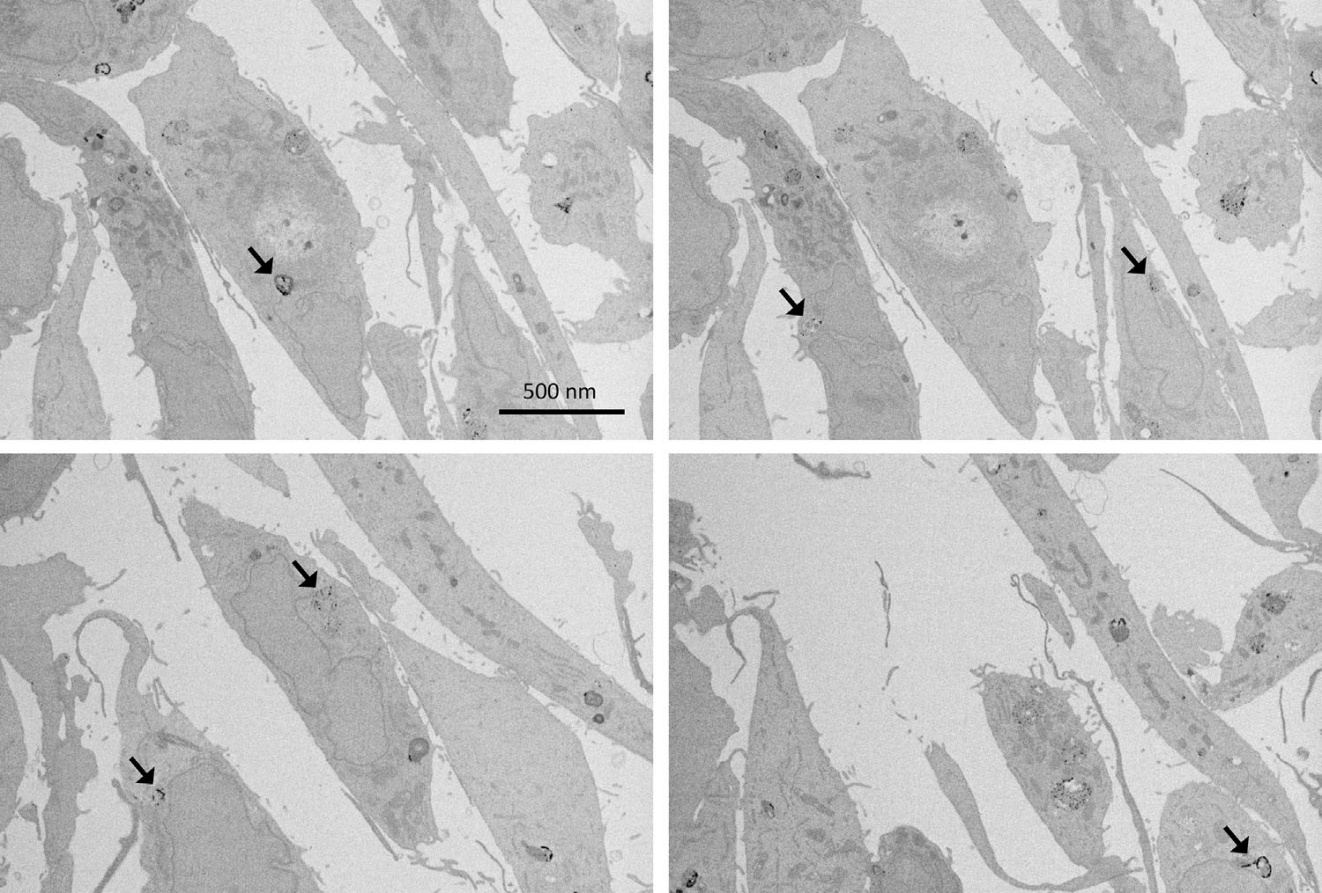
Scanning electron microscopy images of MDA‐MB231 cells treated with 200 μg/mL AuNPs 15 nm for 24 h. Arrows indicate AuNPs clusters. Section thickness: 40 nm

To better characterize the localization of the internalized AuNPs, various Electron Microscopy (EM) analyses were applied. SEM analyses of MDA‐MB‐231 cells treated with 200 μg/mL (9.7 nM) AuNPs 15 nm for 24 h were conducted using a VolumeScope SEM. Serial 40‐nm‐thick sections were cut and imaged, allowing for a full 3D reconstruction of the sample, available as a video file in the Supplementary Materials section (VideoS2.avi). As demonstrated also by the representative frames reported in Figure 4, SEM analysis confirmed the presence of AuNPs clusters (arrows) in the perinuclear region, approximately at 10 nm distance from the nucleus. No AuNPs were present in the nucleus.

**FIGURE 5:**
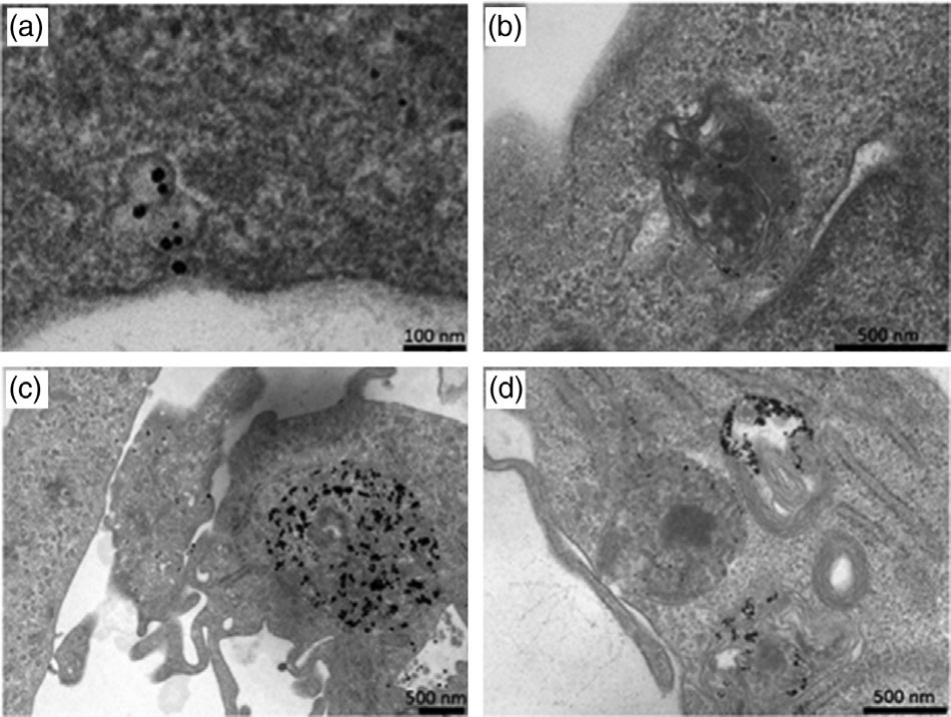
Transmission electron microscopy images of MDA‐MB‐231 cells treated with 200 μg/mL AuNPs 15 nm for 6 h (a, b) or for 24 h (c, d). 60‐nm thick cell sections.

For a more detailed analysis of AuNPs clusters (i.e., groups of large numbers of closely packed AuNPs), we also performed Transmission Electron Microscopy (TEM) on MDA‐MB‐231 cells treated with 200 μg/mL (9.7 nM) AuNPs 15 nm, for 6 and 24 h. TEM analysis at 6 h (Figure 5a) shows AuNPs engulfed by cell membrane and endocyted into vesicles. Further investigation is needed to better characterize the uptake mechanisms. Some AuNPs were evidenced both at 6 and 24 h in vesicles morphologically recognized as endosomes (A) or heterophagic structures (B, C). In some sections, AuNPs were arranged in a row‐like pattern (D). Also for TEM imaging, no AuNPs were observed in the cell nucleus.

### 3.4 Clonogenic assay

**FIGURE 6:**
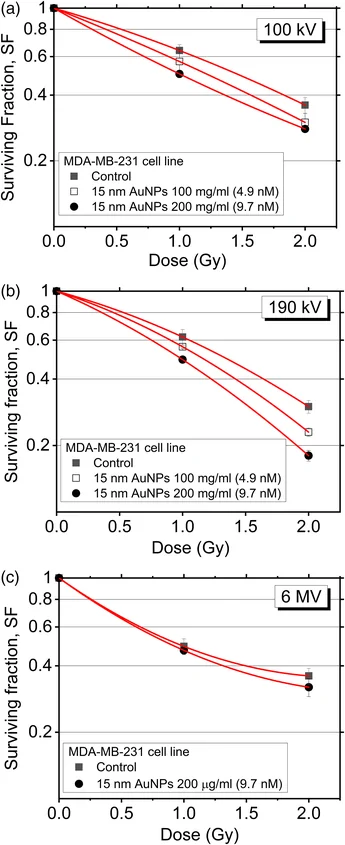
MDA‐MB‐231 cells surviving fraction versus dose. Cells were treated with different 15 nm Aurovist™ AuNPs concentrations (0, 100, 200 μg/mL) and irradiated at (a) 100 kV, (b) 190 kV, or (c) 6 MV. Error bars represent standard errors from triplicate measurements.

We tested, through cell‐survival curves construction, the ability of a single cell of being clonogenic, that is, the ability of a single cell to divide and give rise to a cell colony (at least 50 cells). The loss of this ability as a function of absorbed radiation dose is described by the dose‐survival curve. Figure 6a and b shows the dose‐survival curves for MDA‐MB‐231 cells treated for 24 h with 0, 100, or 200 μg/mL (0, 4.9, and 9.7 nM, respectively) AuNPs 15 nm, and subsequently irradiated with kilovoltage X‐rays at 100 or 190 kV, at doses of 1  or 2 Gy. For MV irradiation, Figure 6c shows the dose‐survival curves for 0 and 200 μg/mL (9.7 nM) AuNPs 15 nm irradiated with 6 MV X‐rays. In terms of *DEF*_40%_ (the ratio of the dose needed to obtain a 40% *SF* with radiation only, to the dose needed to obtain the same *SF* with the same irradiation plus AuNPs), results are shown in Table 1. We also evaluated the *MID* and the *SER* parameters. As shown in Table 1, for kV irradiation, the *DEF*_40%_ increased with increasing AuNPs concentration. At 200 μg/mL AuNPs concentration, the *DEF*_40%_ at 100 and 190 kV is higher than that at 6 MV. These findings are consistent with the interpretation of the higher efficacy of photoelectric absorption in producing a largely significant radiation enhancement in MDA‐MB‐231 breast cancer cells, particularly, for photon energies higher than the K‐edge of Au at 80.7 keV. This is confirmed by the fact that one can see no significant differences between the values obtained at 100 and 190 kV. It is possible to derive similar conclusions if we look at the values of all investigated parameters (*SF*, *MID*, and *SER*) reported in Table 1. In the case of irradiation with 6 MV photons, we noted no influence of the presence of AuNPs.

**TABLE 1:** Values of *SF*_2_ (Surviving Fraction at 2 Gy), *MID*, *SER*, and *DEF*_40%_ (dose enhancement factor at 40% surviving fraction) for MDA‐MB‐231 cells treated at two concentrations of 15 nm Aurovist™ AuNPs and three qualities of X‐rays

X‐ray beam quality	15 nm AuNP concentration	*SF*_2_	*MID*	*SER*	*DEF*_40%_
100 kV	0 μg/mL	0.36 ± 0.03	1.26	1.00	
	100 μg/mL (4.87 nM)	0.30 ± 0.03	1.16	1.10	1.17 ± 0.08
	200 μg/mL (9.74 nM)	0.28 ± 0.01	1.05	1.20	1.34 ± 0.02
190 kV	0 μg/mL	0.30 ± 0.02	1.26	1.00	
	100 μg/mL (4.87 nM)	0.23 ± 0.01	1.16	1.08	1.15 ± 0.06
	200 μg/mL (9.74 nM)	0.18 ± 0.01	1.05	1.20	1.33 ± 0.06
6 MV	0 μg/mL	0.36 ± 0.04	1.10	1.00	
	200 μg/mL (9.74 nM)	0.32 ± 0.03	1.06	1.04	1.14 ± 0.06

## 4 DISCUSSION

Gold‐based nanoenhancers showed promising efficacy against cancer in both in vitro and in vivo systems. Since the pioneering in vivo study, in 2000, where Herold et al.^16^ first reported that gold microspheres could produce biologically effective dose enhancement for kilovoltage X‐rays, the field has seen a proliferation of studies that systematically confirmed the radioenhancement property of AuNPs. Those authors injected intravenously increasing concentrations of 1.9 nm AuNPs (from 0 to 2.7 g Au/kg body weight) in mice carrying EMT‐6 subcutaneous mammary carcinomas, treated with 26 Gy of 250 kVp X‐rays. To date, there have been several hundred articles published on AuNPs radiation sensitization and to make a meaningful comparison among studies is problematical because particle and radiation parameters, as well as cell type, are very diverse.

A common focus of in vitro studies for nanoradioenhancement at subcellular level is the detailed description of nanoparticle internalization. AuNPs increase the radiation energy deposition process locally. While the main target of ionizing radiation damage is cellular DNA in the nucleus, the localization of AuNP particles in the nucleus may not represent a crucial factor for effective cell damage. Indeed, a significant radio‐sensitization effect has been observed for AuNPs localized outside the nucleus.^40^ Several groups^39^, ^40^, ^41^, ^42^ have demonstrated AuNPs localization in vesicles, autophagosomes, and lysosomes. As demonstrated by Yang et al.,^43^ it is noteworthy that the not‐functionalized AuNPs are unable to enter the nucleus as the importation through the nuclear pores necessarily requires recognition sequences.

In this work, we treated MDA‐MB‐231 breast cancer cells with 1.9 and 15 nm AuNPs. After 24 h from incubation, the concentration of 15 nm AuNPs in the cells, measured by atomic absorption spectroscopy, was higher than with 1.9 nm AuNPs. This gives an indication that 15 nm AuNPs may preferentially accumulate in MDA‐MB‐231 breast cancer cells, better than smaller AuNPs. A different internalization of AuNPs based on shape and size has already been demonstrated.^42^, ^44^ The concentration of Au that we measured in treated cells is in the range of μg/mg, which is similar to the concentration of Au observed in tumor tissues (7 mg Au/g tumor tissue) after in vivo AuroVist™ administration.^13^ It is hard to compare in vitro and in vivo concentrations, however, the fact that the order of magnitude is the same suggests that our in vitro model in this aspect could be representative of in vivo conditions. For this reason, subsequent experiments were carried out only with 15 nm AuNPs, which demonstrated also no toxicity up to 400 μg/mL (19.5 nM).

To characterize AuNPs internalization, we performed an overtime experiment using a time‐lapse technique. The video showing the events for as long s 16 h, following MDA‐MB‐231 cells treatment with 200 μg/mL (9.74 nM) AuroVist™ 15 nm, clearly confirmed that AuNPs enter into the cells and localize in cytoplasm but are not able to enter into the nucleus. From the video, it is also evident that AuNPs inside cytoplasm aggregates forming clusters of different sizes. Furthermore, it also shows that AuNPs clusters, the largest of which appear to be enveloped by membranous structures, move within the cytoplasm localizing for a certain time in the perinuclear region. In dividing cells, AuNPs appear to split between daughter cells, suggesting that daughter cells may also be promptly sensitive to radiation thanks to directly inherited AuNPs. This observation points toward including cell internalization dynamics in future models of AuNP radio‐sensitization, since AuNPs accumulation in cells due to uptake from the cellular environment might not represent the unique pathway to gold intake in highly duplicating cancer cells.

To further investigate AuNPs internalization, we analyzed MDA‐MB‐231 cells treated with 200 μg/mL AuNPs by SEM and TEM. These techniques confirmed that AuNPs are internalized in clusters in double membrane‐delimited vesicles, sometimes close to the nucleus. Double membrane vesicles need further investigation to be characterized as autophagolysosomes or autophagosomes. TEM images highlight the interaction of the AuNPs with the cell membrane and their internalization by endocytosis pathway. No AuNP was visible in the cell nucleus during the whole experimental sessions.

The same MDA‐MB‐231 cellular line was previously investigated by other groups under irradiation with 225 kVp X‐rays^16^: after incubation with 1.9 nm AuNPs (500 μg/mL), a *DEF* of 1.23 was observed,^16^ at a dose of 2 Gy. For the same cell line treated with 12 μM AuroVist™ 1.9 nm AuNPs (equivalent to a concentration of 500 μg/mL) and irradiated with 160 kVp and 6 MV photons, the radiation *SER* obtained was 1.41 and 1.29, respectively.^45^ In this work, we have irradiated MDA‐MB‐231 cells treated with 15 nm AuNPs at 2.5 to 5 times lower concentrations (i.e., 100 and 200 μg/mL) than those in previous studies, with X‐rays at 100 and 190 kV and 6 MV beam energy, with *SER* values of 1.20 at kV energies and 1.04 at 6 MV. The 100 and 190 kV energies were chosen to verify a potential dose enhancement effect due to the different average energies of the spectra (43 keV for 160 kV X‐rays, and 81 keV for 190 kV X‐rays) near to, respectively, the L‐edge and the K‐edge of gold.

After all irradiations, at doses up to 2 Gy, cell‐survival curves showed increased mortality of cells (evaluated in terms of *SF*, *MID*, *SER*, and *DEF* parameters) when incubated with 15 nm AuroVist™ AuNPs, with respect to irradiation without AuNPs. In particular, the cell mortality increases with increasing AuNPs concentration. The highest effect of radioenhancement by AuNPs was observed for a kV spectrum (rather than for a MV spectrum) and 200 μg/mL (9.74 nM), where the measured *DEF*_40%_ for MDA‐MB‐231 cells incubated with 15 nm AuNPs was 1.33 ± 0.06. Correspondingly, at 6 MV, the *DEF*_40%_ was 1.14 ± 0.06.

The above findings (specifically at 6 MV) might find confirmation and some understanding in terms of the recent literature on in silico studies,^46^, ^47^, ^48^ as refers to 15 nm AuNPs, with the distinction that the observed *DEF* values are in line with the available literature, but they were obtained at lower AuNPs concentrations. Future work in our new kV breast RT project will show if higher concentrations in vivo produce significantly higher *DEF* and *SER* values at MV as well as kV photon energies.

We have chosen AuroVist™ AuNPs following a series of in vivo studies conducted with mouse mammary subcutaneous tumours.^7^, ^13^ These AuNPs were used as X‐ray contrast agent for microCT, an application of renovated interest in X‐ray imaging.^49^ We proposed to perform in vivo studies to characterize AuNPs localization, also in low vascularized tumors.

Finally, our still‐ and time‐resolved cell microscopy observations point toward a very significant presence of AuNPs clustering in cellular vesicles, where AuNPs clusters form and disrupt with a time scale in the order of tens of seconds: an effect which deserves further investigation on the radio‐sensitization efficiency and possible shielding of low‐energy secondary electrons, for a given AuNPs concentration in the cell culture medium.^48^

## CONFLICT OF INTEREST

The authors declare no conflict to interest disclose.

## FUNDING INFORMATION

This work was, in part, funded by the Italian National Institute for Nuclear Physics (INFN) under the project SR3T.

## Supporting information

Video file for optical holotomography: mp51348‐sup‐0002‐VideoS1.avi

Video file for SEM imaging: mp51348‐sup‐0002‐VideoS2.avi

## Data Availability

Data available in article supplementary material.
